# Learning from communication versus observation in great apes

**DOI:** 10.1038/s41598-022-07053-2

**Published:** 2022-02-21

**Authors:** Hanna Marno, Christoph J. Völter, Brandon Tinklenberg, Dan Sperber, Josep Call

**Affiliations:** 1 Department of Cognitive Science, Central European University, Budapest, 1051 Hungary; 2 Department of Cognitive Psychology, Eötvös Lóránd University, Faculty of Education and Psychology, Budapest, 1064 Hungary; 3 Messerli Research Institute, University of Veterinary Medicine Vienna, Medical University of Vienna, University of Vienna, 1210 Vienna, Austria; 4 Department of Philosophy, York University, Toronto, ON M5R 2M8 Canada; 5 School of Psychology and Neuroscience, University of St Andrews, St Andrews, KY16 9JP UK; 6 Max Planck Institute for Evolutionary Anthropology, 04103 Leipzig, Germany

**Keywords:** Psychology, Human behaviour, Cultural evolution, Animal behaviour

## Abstract

When human infants are intentionally addressed by others, they tend to interpret the information communicated as being relevant to them and worth acquiring. For humans, this attribution of relevance leads to a preference to learn from communication, making it possible to accumulate knowledge over generations. Great apes are sensitive to communicative cues, but do these cues also activate an expectation of relevance? In an observational learning paradigm, we demonstrated to a sample of nonhuman great apes (bonobos, chimpanzees, orangutans; N = 24) how to operate on a food dispenser device. When apes had the opportunity to choose between an effective and an ineffective method in the baseline conditions, the majority of them chose the effective method. However, when the ineffective method was demonstrated in a communicative way, they failed to prioritize efficiency, even though they were equally attentive in both conditions. This suggests that the ostensive demonstration elicited an expectation of relevance that modified apes’ interpretation of the situation, potentially leading to a preference to learn from communication, as human children do.

## Introduction

Seeking eye-contact or calling the other’s name are typical actions we use in order to initiate communicative interactions with others. These signals, also called ostensive cues^1^, can inform the addressee about our intention to convey to them something that is relevant enough to them to be worth their attention^2^. The communicator’s capacity to produce ostensive cues together with the audience’s matching disposition to expect what is ostensively communicated to be of particular relevance to them can be a crucial component in social interaction. It allows one individual to influence the mental states of others by not only drawing their attention to some items in the common environment but also by causing others to interpret these items as more relevant than they would have done if their attention had been attracted to these items in a non-ostensive way.

In the past decade, various studies provided evidence that human infants, immediately after birth, are ready to interpret some of these signals (such as eye-contact or infant-directed speech) as indicating communicative intentions^3–5^, and that this sensitivity to communicative signals is found across different cultures^6^. Infants do not only orient towards the sources of these signals, but they also actively search for further referential cues in order to identify the content of the communication, for instance they can infer the presence of hidden objects from referential gaze information^7^. Furthermore, already at the age of 18 months they interpret new information in the context of ostensive cues as relevant to them, therefore worth acquiring^8^.

As in human communication, there is evidence that under certain circumstances great apes can also produce and react to ostensive cues as signals of communicative intentions. For example, while eye-contact can be a sign of potential threat or attack in the animal kingdom, apes seem to use eye-contact also as an expression of their intention to communicate with others^9^. Hand-reared gorillas tend to make eye-contact with their caregivers in order to direct their attention before making a request regarding a certain object or location^10^, and chimpanzees living in captivity use eye-contact and gaze alternations to objects during their interactions with conspecifics and humans^11,12^. Similar to chimpanzees, bonobos and orangutans also seem to use eye-contact, gaze alternations, and imperative gestures both with their conspecifics and humans^13–16^. Furthermore, similar to human infants, captive apes can be sensitive to communicative cues, such as eye-contact or calling their name^17–19^, and they also prefer direct eye-gaze over averted gaze^20^.

However, it remains unclear how apes interpret the information they receive in the context of ostensive signals. In the case of infants and young children, intentionally communicated information can activate an expectation of relevance, i.e. that the new information is relevant for them, therefore worth acquiring. For example, when young children observe action demonstrations, even if the communicatively demonstrated action is apparently not the most effective way to achieve the intended outcome, children tend to copy it^21^. In a recent study, when 18-month-old infants observed two models operating differently on an unfamiliar device, they tended to repeat the action of the demonstrator who made an eye-contact with them and talked to them in infant-directed speech, and not the action of the demonstrator who provided no such communicative signals, even though the non-communicative demonstrator managed to achieve the expected outcome more frequently than the communicative one^22^. Thus, in the case of infants and young children, it seems that the attributed relevance of an ostensively presented action can override perceived efficiency.

There is exhaustive evidence that great apes are also able to acquire novel information via observing the other’s behavior^23–27^, Myowa-Yamakoshi and Matsuzawa ^28^. However, is it possible that similar to humans, apes perceive the communicatively demonstrated information as relevant, therefore worth copying, even at the expense of its efficiency? Considering that apes seem to show similar sensitivity to ostensive signals as human infants, it is possible that they also perceive the ostensively demonstrated information as being more relevant, which might lead to selective copying, similar to human infants. The current study aimed at investigating the hypothesis whether, similar to human infants, nonhuman great apes would also attribute relevance to the ostensively presented information. In an observational learning paradigm, we manipulated two factors: the efficiency of the demonstrated actions and the use of ostensive signals vs. some non-social attention-getter cues by the demonstrators (in order to be sure that apes would pay equal attention in all conditions). Since there is evidence that apes can successfully detect and copy the more effective method^29^, we put the efficiency of the method in conflict with the use of ostensive signals when demonstrating to apes how to operate a food dispenser device. If apes do not consider the ostensively demonstrated information as being more relevant to them, we predicted, they should copy the more effective method, irrespective of ostensive cues. However, if ostensive could trigger an expectation of relevance also in apes, then they should stop prioritizing efficiency when it is in conflict with the use of communicative cues.

## Methods

A total of 24 great apes (6 bonobos, *Pan paniscus*, 12 chimpanzees, *Pantroglodytes*, 6 orangutans, *Pongo abelii*) participated in this study (Supplementary Table S1). They were born in captivity and lived with conspecifics in enriched naturalistic environments at the Wolfgang Kohler Primate Research Center (WKPRC) in Leipzig, Germany. The study was approved by the joint ethical committee of the Max Planck Institute for Evolutionary Anthropology (Leipzig, Germany) and Leipzig Zoo. The apes were neither food nor water-deprived and could participate or refuse to participate in the study by their own choice. Animal husbandry and all research methods complied with local guidelines, which strictly adhere to international standards [the Weatherall report “The use of non-human primates in research”] and the national laws of Germany [“EAZA Minimum Standards for the Accommodation and Care of Animals in Zoos and Aquaria”, “WAZA Ethical Guidelines for the Conduct of Research on Animals by Zoos and Aquariums”, “Guidelines for the Treatment of Animals in Behavioral Research and Teaching” of the Association for the Study of Animal Behavior (ASAB)”].

Apes watched two demonstrators operating a food dispenser. The dispenser could be activated by inserting certain objects. Each trial started with four demonstrations in sequence, two performed consecutively by each demonstrator and each of the demonstrators performing the same action, but using different objects each time to activate the device. After observing the demonstrations, apes had the opportunity to choose between the two objects and try to activate the device by inserting the chosen object into the device.

Apes participated in three conditions, and each condition consisted of two trials. The procedure followed the original design that was used both in the study of Gopnik et al.^30^ and Völter et al.^29^. Our previous results indicated that two demonstrations were sufficient for the apes to distinguish between the objects’ effects on the apparatus^29^.

Apes participated in the three conditions sequentially in the following order: first they attended the Only Ostensive Condition, then the Only Non-Ostensive Condition, and finally the Ostensive vs. Effective Condition. The different conditions were tested on average with a 1.5-month long time gap in between two consecutive conditions, to mitigate the risk for carry-over effects across conditions.

In every trial, apes were presented with a novel pair of objects. In the Only-Ostensive condition both demonstrators used ostensive cues, but while one of them managed to activate the device twice (i.e. every time) when inserting the object, the other demonstrator failed in both attempts with a different object. During this Only-Ostensive condition, the demonstrator first made eye-contact with the subject, said ‘hello’ while holding the object so that it was visible to the ape. Then she/he approached the device, said ‘hello’ again, clapped with her hands and showed the object again before inserting it into the device. We decided to use these cues because they have been considered as signals indicating communicative intention for the apes^9,31,32^. The demonstrator started to operate on the device only when the ape was attentive and could see the entire action. In the case of effective demonstrations, once the demonstrator had inserted the object, the device gave a short tone (this was controlled by the demonstrator, using a foot pedal hidden from the view of the ape), accompanied by flashing lights and the release of a small food pellet accessible to the apes. During the ineffective trials, on the other hand, after the demonstrator had inserted the object nothing happened to the device nor did the ape get any reward. After completing the four demonstrations (two successful and two unsuccessful), the two objects were placed on a tray and pushed on the two sides by the two demonstrators towards the subject (at this point none of them made eye-contact with the ape) so that she/he could choose between the two objects and operate on the device by herself/himself. Each demonstrator sat behind the respective object that they had inserted during the demonstration phase. Apes’ actions (object insertions) were rewarded by a food pellet released from the device only if they chose the effective object. The Only-Ostensive condition served as a baseline to see whether apes would reliably choose the object that was successfully used by one demonstrator, as opposed to the object used by the other demonstrator and that had never worked.

In the Only-Non-Ostensive condition, on the other hand, neither of the demonstrators communicated with the subjects, but again one of them managed to activate the device twice, whereas the other demonstrator failed on both attempts. In this condition, both demonstrators first held the object in such a way that it was visible to the ape but, instead of making eye-contact with the subject, they only looked at the object and said ‘aha’. Then they approached the device, held again the object in a way that made it visible to the ape and said ‘aha’. Before inserting it into the device, they knocked the floor twice with the object. These actions served to elicit the same amount of attention from the apes as in the Ostensive condition, but did not include any communicative gestures. The rest of the Only-Non-Ostensive condition was the same as in the Only-Ostensive condition. This condition allowed us to compare the two conditions in order to see whether the presence of communicative cues would modify apes’ performance and, more specifically, would affect their ability to choose the effective object to operate on the device.

**Figure 1:**
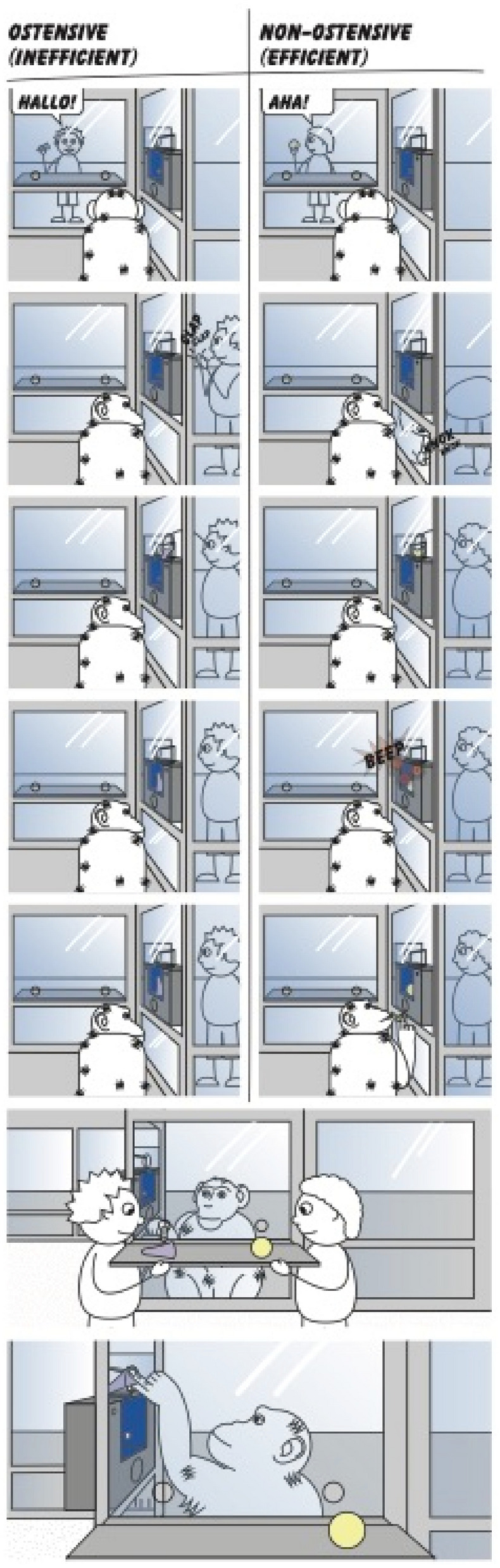
Schematic representation of the Ostensive vs. Effective condition. During the Ostensive (ineffective) demonstration the demonstrator provided communicative cues to the ape, but failed to activate the device. During the Non-Ostensive (effective) demonstration the demonstrator did not communicate with the ape, but successfully activated the device. After the demonstrations, the ape had the opportunity to choose between the two objects and to try to activate the device.

Finally, in the Ostensive vs. Effective condition we pitted the presence of communicative cues against efficiency. For this, one demonstrator was effective on both trials without communicating with the apes and only using those non-social attention getter cues that were used in the Only-Non-Ostensive condition, whereas the other demonstrator, who was communicating with the apes failed to activate the device on both trials. This condition aimed at testing whether apes, similar to children, would rely more on the communicatively presented demonstration than on their own observation of efficiency (Fig. 1).

Subjects participated in two trials in each condition. Each trial consisted of two demonstrations, performed by two demonstrators. While one demonstrator was performing her demonstration, the other demonstrator was standing in the corner of the testing room. Out of the two trials, one trial always started by a successful demonstration, followed by an unsuccessful demonstration, whereas the other trial started by an unsuccessful demonstration, followed by a successful one. The order of the successful vs. unsuccessful demonstrations was counterbalanced. Each trial ended by the object choice of the subject.

In total, five demonstrators (two females and three males) provided the demonstrations across the different conditions. The demonstrators were counterbalanced in such a way that for each subject they performed the same communicative role (i.e. they were consistently either communicating or not across the different conditions).

## Results

**Figure 2:**
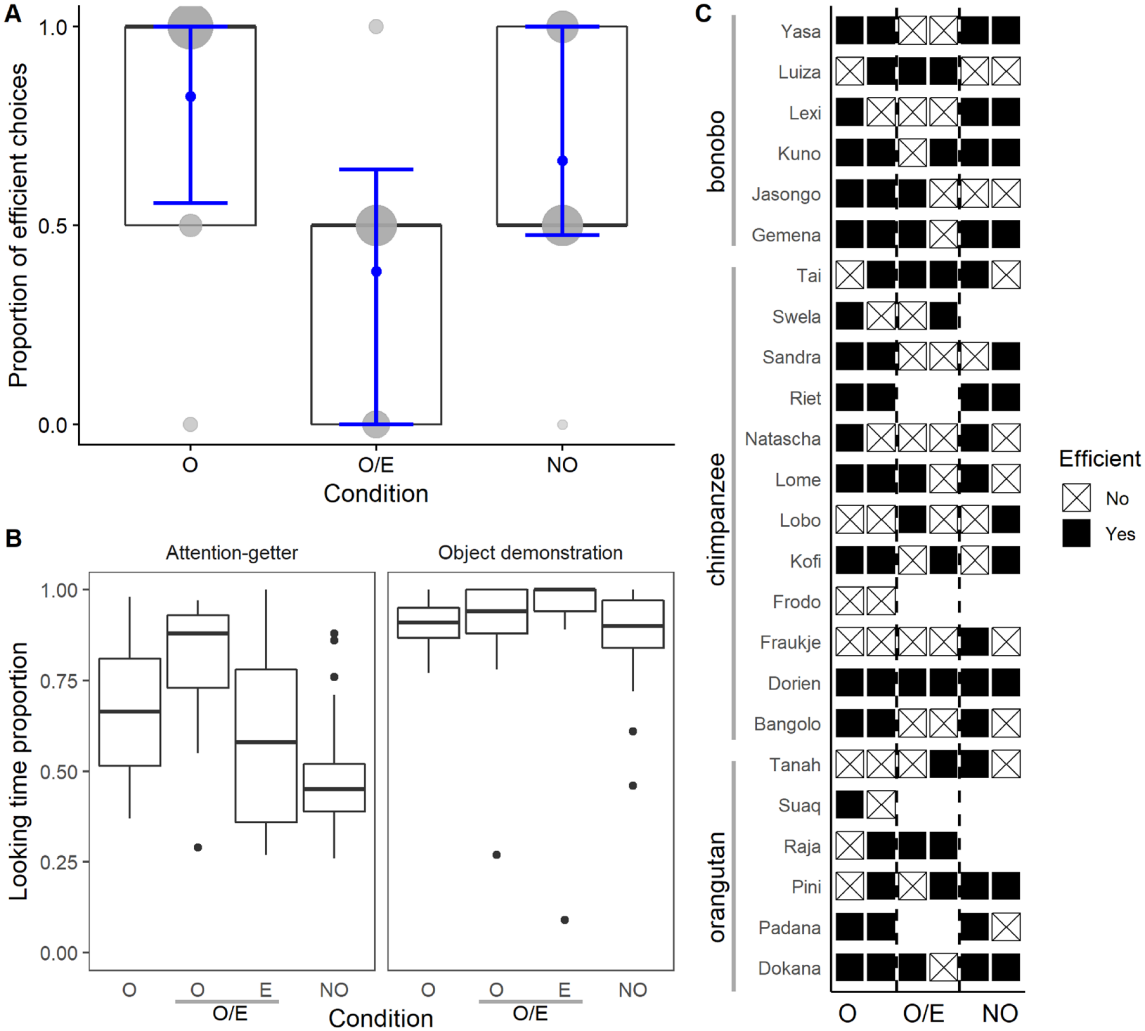
The apes’ performance across the three conditions. (**A**) Box plot depicting the performance of individuals who completed all three conditions with the fitted model (blue dot) and the 95% confidence intervals (blue whiskers). The center line of the box shows the median, the box limits the upper and lower quartiles. The grey dots show the individual performance, the size of the dots is proportional to the number of represented individuals. (**B**) Box plot of apes looking times during the presentation of the attention-getters and during the object demonstration across conditions (center line, median; box limits, upper and lower quartiles; whiskers, largest/smallest value within the 1.5 × interquartile range; points, outliers). (**C**) Individual scores in each condition (filled squares: effective object choices, squares marked by a cross: ineffective object choices).

Apes’ object choices were recorded (see the data in the Supplementary Data File). First, we calculated the percentage of successful object choices (i.e. when the ape chose the effective object). In the Only-Ostensive condition, we obtained data of 24 subjects (12 chimpanzees, 6 orangutans and 6 bonobos). During this condition, the effective object was chosen on 67% of the trials, which was significantly better than expected by chance (one-sample t-test: t(23) = 2.14, p = 0.043, 95% CI [0.506, 0.827]). In the Only-Non-Ostensive condition, we collected data of 20 subjects (10 chimpanzees, 4 orangutans and 6 bonobos). During this condition apes chose the effective object on 65% of the trials (t(19) = 2.04, p = 0.055, 95% CI [0.496, 0.804]). However, in the critical Ostensive vs. Effective condition, where we collected data of 20 subjects (10 chimpanzees, 4 orangutans and 6 bonobos), apes chose the effective object only on 45% of the trials, which was not significantly different from chance level (t(19) = − 0.62, p = 0.541, 95% CI [0.282, 0.618]) (Fig. 2C). Furthermore, when we compared the performance of individuals who completed all three conditions in a binomial GLMM (N = 18; proportion of variance in the response explained by the model: conditional R^2^ = 0.45; see ESM for details), we found no evidence that the species’ performance differed across conditions (condition: species interaction: χ^2^ = 3.32, df = 4, p = 0.505). We then fitted a reduced model without the interaction term to examine the main effects. We found that apes’ performance varied significantly across conditions (χ^2^ = 7.19, df = 2, p = 0.027; Supplementary Table S2). They chose the effective object less often in the Ostensive vs. Effective condition than in the Only-Ostensive condition (χ^2^ = 5.09, df = 1, p = 0.024; see Fig. 2A). We found a similar, though not significant, difference between the Ostensive vs. Effective and the Only-Non-Ostensive condition (χ^2^ = 3.01, df = 1, p = 0.083). The trial number within each condition (χ^2^ = 0.63, df = 1, p = 0.429) and species (χ^2^ = 1.02, df = 2, p = 0.600) had no significant effect on the performance.

In a secondary, exploratory analysis, we also added rearing history to the model and the interaction between rearing history and condition. However, neither the interaction (χ^2^ = 3.16, df = 2, p = 0.206) nor the main effect of rearing history (in a reduced model without the interaction term) were significant (χ^2^ = 1, df = 1, p = 0.703; for the model results, see Supplementary Table S3). In another exploratory analysis we investigated whether adding the identity of the effective experimenter as a random effect would improve the model fit but found no evidence for this (the model without the additional random intercept of experimenter ID had a lower AIC: ΔAIC 2.0; see Supplementary Table S4 for the model results).

Finally, we measured apes’ looking time in each condition, separately during the presentation of the communicative or non-social attention getter cues, and during the action demonstrations. We decided to do this because even though in the Non-Ostensive trials we never started the demonstration before the experimenter was certain that the apes were paying attention, we wanted to exclude any possibilities that the effect we found could have been due to differences in apes’ attention. We found that during the presentation of the communicative and the non-social attention getter cues, apes looked significantly more at the communicative demonstrator than at the non-ostensive demonstrator both in the Ostensive vs. Effective condition (t(20) = 4.737, p < 0.001, Bayes Factor for the alternative vs. the null hypothesis: 223.120) and when comparing the Only-Ostensive condition to the Only-Non-Ostensive condition (t(20) = 5.208, p < 0.001, BF: 592.687). During the object demonstration, however, we found no significant difference in looking time between the conditions (t(20) = − 1.895, p = 0.073, BF: 1.022; t(20) = 1.669, p = 0.111, BF: 0.745) (Fig. 2B). Thus, apes’ object choices did not reflect increased attention to the communicatively performed action demonstrations.

## Discussion

Our results suggest that similar to young children, apes can also be influenced by the ostensive cues of the demonstrator. In the baseline conditions, when communicative signals were not in conflict with efficiency, the majority of them chose the effective object more often than the ineffective one (though the comparison to the hypothetical chance level failed to reach significance in the non-ostensive condition). However, when we pitted efficiency against communication, apes stopped prioritizing efficiency and chose the ineffective, but communicatively demonstrated object equally often as the effective one. Even though the number of ineffective object choices was not significantly different from the hypothetical chance level in the Ostensive vs. Effective condition, the fact that in comparison with the baseline conditions apes clearly changed their behavior indicates that ostensive cues modified their initial preference towards the effective objects.

A limitation of our study is the limited sample size and the resulting moderately powered analyses, compared to studies conducted with human subjects. The small sample size also limits the conclusions that can be drawn concerning potential species differences and the lack of evidence for such differences should be interpreted with caution. In addition, while we replicated here the principal finding of the study of Völter and Call ^29^ (i.e., above-chance performance after witnessing limited evidence for the efficiency of novel objects), just like in the original study, the performance in this observational learning task did not lead to large effect sizes. Thus, the small effect sizes and the limited sample size led to marginally significant effects for the comparisons between the different conditions.

One might also point out that since apes participated in the three conditions in a fixed order, this could have potentially affected their performance (for example leading to fatigue by the time they participated in the third condition). While we acknowledge that assigning them randomly to the three conditions would have been favorable, logistically this would have raised some difficulties, since we were careful not to present the same demonstrator in different (i.e. ostensive vs. non-ostensive) roles. However, the fact that the different conditions were tested on average with a 1.5 month long time gap in between two conditions, and one testing session did not last longer than 4–5 min, makes it unlikely that apes would have become tired or less motivated by the time they participated in the third condition.

Another limitation that we should mention here concerns in general the limitation of the observational learning paradigm we used in the study. While these paradigms usually give evidence about preferences to copy certain models or types of behavior, it is rarely tested whether these preferences would have an effect also on long-term memory processes, i.e. whether subjects would encode the copied behavior in a long-term. Therefore, although from the study we can draw the conclusion that great apes, similar to human infants, tend to interpret the information communicated as being relevant to them, we cannot tell whether this attribution of relevance would also modify what apes would learn on a long term from communicatively transmitted information. Testing this question should be a next step in the investigation of apes’ social learning processes.

Traditional notions of stimulus/local enhancement^33^ do not satisfactorily explain subjects’ choices in the ostensive vs. efficient (non-ostensive) condition because subjects watched the object demonstrations equally in both conditions. Similarly, the notion of social enhancement does not fully capture the phenomenon we described here because even though subjects paid more attention in the communicative phase of the ostensive compared to the efficient (non-ostensive) condition (which is consistent with social enhancement), such attentional differences disappeared during the object demonstration phase. If social enhancement were the only process at work here, one would have expected that differential attention would have also been apparent during the object demonstration phase, which is precisely what one sees in cases of social learning strategies based on following dominant or prestigious individuals^34^.

One might argue that the experience of our zoo-housed apes with human communicative signals could trivially explain the current findings: apes might have associated certain communicative signals with food rewards. However, the ostensive cues that we used in the present studies (hand-clapping and saying ‘hello’) were clearly distinct from the gestures and utterances that the caretakers typically use (i.e. calling the apes by their names to initiate interactions). Additionally, the cues zookeepers tend to use in order to signal feeding-time for the apes are usually related to the food itself (for example shaking a bucket containing the food), while in our case the demonstrators never performed any actions in relation with the food reward.

Another possibility, however, is that the ostension-induced attentional processes indeed affected apes’ memory, but only for the ostensively highlighted object, and not for the action demonstration and the subsequent outcome (i.e. being rewarded or not). Such decoupling of the object from the action demonstration would be somewhat surprising; nevertheless we cannot exclude this possibility. However, if ostensive cues could potentially increase the saliency of the highlighted object, leading to a better encoding of it, shouldn’t we consider this mechanism as an attention-related precursor of relevance attribution for the ostensively presented information?

Recent evidence seems to support the hypothesis that apes might react to ostensive cues with an enhanced attention and exploratory behavior. In the study of Kano and his colleagues (2018) chimpanzees watched an experimenter, who either made eye-contact with them and called their name (in the Ostensive condition), or was shown together with a salient, attention-grabbing cue (in the Control conditions) and then gazed towards one of two objects. Even though chimpanzees spent equal time with looking at the experimenter’s face in both conditions, when the experimenter first provided them ostensive signals, they tended to spend more time by looking at both objects in the ostensive condition. This indicates that even though they failed to understand what was the intended referent of the communicative signals (i.e. the gazed at object), ostensive cues still elicited an increased interest and attention, leading to longer exploration of the scene (Kano et al. ^35^), compared to the other non-communicative attention-grabbing signals.

It is a well-established fact that, from a very early age, humans are able to interpret ostensive cues as indicators of relevance (e.g. Refs.^8^, Kiraly et al. 2013). The main finding of our study is that, similarly, apes seem to be capable of interpreting the same objects or events differently depending on whether they are ostensively presented to them or not. When apes are presented two objects in the same manner (either both ostensively or both non-ostensively) with evidence that one of the two objects can be used effectively to obtain a reward and the other cannot, they tend to choose the effective object. When, however, the ineffective object is presented ostensively and the effective object non-ostensively, this preference for the effective object disappears. This suggests that the ostensive highlighting of the object can modify its saliency, leading to a change in apes’ initial preferences, which would be otherwise solely based on the efficiency of the objects. We propose that this effect can be considered as an attention-related precursor of relevance attribution of apes for the ostensively presented information. In other words, our results indicate that similar to human infants, also apes might consider the ostensively demonstrated information as being relevant to them.

If ostension exists among non-human apes, how similar is it to ostension among humans? In an earlier study of Marno and Csibra^22^, 18-month-old infants preferred a less reliable method ostensively presented by one experimenter to a more reliable method non-ostensively presented by another experimenter. This study provided further evidence for the view that, among humans and especially in adult-infant interactions, ostensive cues typically elicit not just any expectation of relevance but the expectation that the objects or events ostensively displayed are relevance because they provide useful general knowledge^36^. According to this theory, human infants might be equipped with a special, innate learning system that helps them to detect ostensive signals from birth^37^. Once these cues were registered, infants can attribute communicative intention to the source of the ostensive cues and make the inference that the person is about to convey some relevant information to them, henceforth important to learn. As a result, they will actively search for further referential cues in order to figure out the content of the new information, which then will be preferentially encoded as some generic, universally shared knowledge^36,37^.

One possibility suggested by our findings is that, also for apes, communication induces modified attentional and encoding processes, which could be considered as precursors of expectations of relevance. In our study, apes were presented with different ‘methods’ (operationalized by the different objects) in order to demonstrate how to operate on a device. It could be that they were not just influenced in their immediate object choices by the ostensively presented demonstration, but that these choices actually reflected an understanding of ‘what is the correct way of making this device work’.

However, the precise origins of this ability are unclear. One possibility is that sensitivity to some forms of ostension is part of the ape natural communicative abilities that they use with conspecifics. According to this perspective, humans and their primate relatives may have been selected to use ostensive cues as a way to modify the intended meaning of their communicative exchanges with conspecifics or simply use them to gauge the likelihood that a particular event might occur. For example, recent evidence suggests that in some populations, chimpanzees in the wild tend to actively facilitate learning by transferring termite fishing probes to less skilled individuals (although whether chimpanzees intentionally communicate in this case to facilitate learning remains an open question)^38^.

Another possibility is that apes have acquired their sensitivity to ostensive cues by interacting with humans. Human-induced effects on apes for some aspects of social cognition have already been described in the literature. For instance, enculturated apes (those raised by humans in the human home) pay much more attention to humans in general, than mother-reared apes, presumably because the former have experienced humans as a valuable source of information^39^. Additionally, apes interacting with humans in laboratory settings acquire human gestures like deictic pointing to communicate with them about external entities^11,40,41^. Also, enculturated apes can be receptive to elements of intentional teaching, when they have been exposed to human-typical social experiences. For example, orangutans, who were raised by humans show better understanding of referential gesturing^14^ and they prefer to spontaneously imitate human models^42^. In a deferred imitation task, both human-reared juvenile chimpanzees and orangutans tended to imitate the action in the absence of any reinforcement, which raises the possibility that they might be intrinsically motivated to imitate the model, similar to human infants and children^43^.

Thus, even though chimpanzees have not been selected to attend to humans or use their gestures to communicate with them, they can readily do so provided the necessary developmental inputs. These two possibilities that represent phylogenetic and ontogenetic influences, are not mutually exclusive. In fact, both are likely to contribute to the development of ostension in humans. But the paucity of ape data in this area prevents us from determining at this time how each of them contributes to the emergence of apes’ sensitivity to ostension.

However, while these studies might indicate that being in close proximity of humans, also apes could acquire new information via teaching, we should still keep in mind that so far we don’t have sufficient evidence to assume that apes in the wild would intentionally teach their juveniles. Therefore, it seems that sensitivity to ostension—and possibly a precursor of relevance attribution regarding the ostensively presented information—per sé is not a sufficient prerequisite of the emergence of intentional teaching. From the learner’s side, one possibility is that while ostensive cues might lead to an increased attention and longer exploration, there is no clear understanding what would be the intended referent of the communication in the case of ambiguity (similar to the findings of the Kano et al. ^35^ study). Therefore, without the capacity of being able to safely rely on the referential cues of the communicator, apes wouldn’t be able to profit intentional teaching either. Another possibility, however, that even if apes are sometimes able to copy the actions of models in the context of communication, they would fail to encode the imitated action on a long term, which might prevent them from real learning. Indeed, so far studies on apes’ imitation only investigated spontaneous imitative behavior, or imitation after a short delay (e.g. Refs.^42,43^).

Thus, future studies should examine to what extent the short-term bias to preferentially copy the communicatively demonstrated information established in our study would also lead to enduring changes in behavior, i.e. to long-term learning processes. If it does, this could open new horizons in the research of apes’ evolutionary shared capacity to learn from teaching, and possibly also about their potential to accumulate knowledge through generations.

## Supplementary Information

Supplementary Information.

## Data Availability

Data available in the Supplementary Information files of this article.

## References

[1] Csibra G, Gergely G (2009). Natural pedagogy. Trends Cogn. Sci..

[2] Sperber, D. & Wilson, D. *Relevance: Communication and Cognition*. (2nd edition with a new Postface, 1995). (Blackwell, 1986).

[3] Cooper RP, Aslin RN (1990). Preference for infant-directed speech in the first month after birth. Child Dev..

[4] Farroni T, Csibra G, Simion F, Johnson MH (2002). Eye contact detection in humans from birth. Proc. Natl. Acad. Sci..

[5] Masataka, N. *The Onset of Language*, vol. 9. (Cambridge University Press, 2003).

[6] Hernik M, Broesch T (2019). Infant gaze following depends on communicative signals: An eye-tracking study of 5-to 7-month-olds in Vanuatu. Dev. Sci..

[7] Csibra G, Volein A (2008). Infants can infer the presence of hidden objects from referential gaze information. Br. J. Dev. Psychol..

[8] Southgate V, Chevallier C, Csibra G (2009). Sensitivity to communicative relevance tells young children what to imitate. Dev. Sci..

[9] Gómez JC, Russon AE, Bard KA, Parker ST (1996). Ostensive behavior in great apes: The role of eye contact. Reaching into Thought.

[10] Gómez, J. C. El desarrollo de la comunicación intencional en el gorila. Unpublished Ph. D. Dissertation, Universidad Autónoma de Madrid. [JCC]. (1992).

[11] Leavens DA, Hopkins WD, Bard KA (1996). Indexical and referential pointing in chimpanzees (*Pan troglodytes*). J. Comp. Psychol..

[12] Tomasello M, George BL, Kruger AC, Jeffrey M, Evans A (1985). The development of gestural communication in young chimpanzees. J. Hum. Evol..

[13] Bard KA (1992). Intentional behavior and intentional communication in young free-ranging orangutans. Child Dev..

[14] Call J, Tomasello M (1994). Production and comprehension of referential pointing by orangutans (*Pongo pygmaeus*). J. Comp. Psychol..

[15] Lucca K, MacLean EL, Hare B (2018). The development and flexibility of gaze alternations in bonobos and chimpanzees. Dev. Sci..

[16] Pika S, Liebal K, Tomasello M (2005). Gestural communication in subadult bonobos (*Pan paniscus*): Repertoire and use. Am. J. Primatol..

[17] Call J, Agnetta B, Tomasello M (2000). Cues that chimpanzees do and do not use to find hidden objects. Anim. Cogn..

[18] Tomasello M, Hare B, Lehmann H, Call J (2007). Reliance on head versus eyes in the gaze following of great apes and human infants: The cooperative eye hypothesis. J. Hum. Evol..

[19] Ueno A, Hirata S, Fuwa K, Sugama K, Kusunoki K, Matsuda G (2010). Brain activity in an awake chimpanzee in response to the sound of her own name. Biol. Lett..

[20] Myowa-Yamakoshi M, Tomonaga M, Tanaka M, Matsuzawa T (2003). Preference for human direct gaze in infant chimpanzees (*Pan troglodytes*). Cognition.

[21] Király I, Csibra G, Gergely G (2013). Beyond rational imitation: Learning arbitrary means actions from communicative demonstrations. J. Exp. Child Psychol..

[22] Marno H, Csibra G (2015). Toddlers favor communicatively presented information over statistical reliability in learning about artifacts. PLoS One.

[23] Carpenter M, Tomasello M (1995). Joint attention and imitative learning in children, chimpanzees, and enculturated chimpanzees. Soc. Dev..

[24] Fuhrmann D, Ravignani A, Marshall-Pescini S, Whiten A (2014). Synchrony and motor mimicking in chimpanzee observational learning. Sci. Rep..

[25] Myowa-Yamakoshi M, Matsuzawa T (2000). Imitation of intentional manipulatory actions in chimpanzees (*Pan troglodytes*). J. Comp. Psychol..

[26] Stoinski TS, Wrate JL, Ure N, Whiten A (2001). Imitative learning by captive western lowland gorillas (*Gorilla gorilla gorilla*) in a simulated food-processing task. J. Comp. Psychol..

[27] Tennie C, Call J, Tomasello M (2012). Untrained chimpanzees (*Pan troglodytes schweinfurthii*) fail to imitate novel actions. PLoS One.

[28] Myowa-Yamakoshi, M., & Matsuzawa, T. Factors influencing imitation of manipulatory actions in chimpanzees (Pan troglodytes). *J. comparative psychology,* **113**(2), 128. (1999). 10.1037/0735-7036.113.2.128 10384721

[29] Völter CJ, Sentís I, Call J (2016). Great apes and children infer causal relations from patterns of variation and covariation. Cognition.

[30] Gopnik A, Sobel DM, Schulz LE, Glymour C (2001). Causal learning mechanisms in very young children: Two-, three-, and four-year-olds infer causal relations from patterns of variation and covariation. Dev. Psychol..

[31] Kalan AK, Rainey HJ (2009). Hand-clapping as a communicative gesture by wild female swamp gorillas. Primates.

[32] Pika S (2008). Gestures of apes and pre-linguistic human children: Similar or different?. First Lang..

[33] Thorpe WH (1956). Learning and Instinct in Animals.

[34] Hoppitt W, Laland KN (2013). Social Learning: An Introduction to Mechanisms, Methods, and Models.

[35] Kano, F., Moore, R., Krupenye, C., Hirata, S., Tomonaga, M., & Call, J. Human ostensive signals do not enhance gaze following in chimpanzees, but do enhance object-oriented attention. *Animal Cognition* **21**(5), 715–728. (2018). 10.1007/s10071-018-1205-z 30051325

[36] Csibra G, Gergely G (2006). Social learning and social cognition: The case for pedagogy. Process. Change Brain Cognit. Dev. Atten. Perform. XXI.

[37] Csibra G (2010). Recognizing communicative intentions in infancy. Mind Lang..

[38] Musgrave S, Lonsdorf E, Morgan D, Prestipino M, Bernstein-Kurtycz L, Mundry R, Sanz C (2020). Teaching varies with task complexity in wild chimpanzees. Proc. Natl. Acad. Sci..

[39] Bering JM (2004). A critical review of the ‘enculturation hypothesis’: The effects of human rearing on great ape social cognition. Anim. Cogn..

[40] Call J, Tomasello M, Russon AE, Bard KA, Parker ST (1996). The effect of humans on the cognitive development of apes. Reaching into Thought.

[41] Leavens DA (2004). Manual deixis in apes and humans. Interact. Stud..

[42] Russon AE, Galdikas BM (1995). Constraints on great apes' imitation: Model and action selectivity in rehabilitant orangutan (*Pongo pygmaeus*) imitation. J. Comp. Psychol..

[43] Bering JM, Bjorklund DF, Ragan P (2000). Deferred imitation of object-related actions in human-reared juvenile chimpanzees and orangutans. Dev. Psychobiol. J. Int. Soc. Dev. Psychobiol..

