# Supplementary material for: Learning from communication versus observation in great apes
Source: Sci Rep. 2022 Feb 21;12:2917. doi: 10.1038/s41598-022-07053-2 (PMC8861107; doi:10.1038/s41598-022-07053-2)
Supplement: Supplementary file 1 — Supplementary Information. [file 41598_2022_7053_MOESM1_ESM.docx]

**Supplementary Information for:**

**Title:** Learning from communication versus observation in great apes

**Authors:** Hanna Marno^1,2^*, Christoph J. Völter^3,5^, Brandon Tinklenberg^4^, Dan Sperber^1^, Josep Call^5,6^

**Affiliations:**

^1^ Department of Cognitive Science, Central European University, Budapest, 1051, Hungary;

^2^ Department of Cognitive Psychology, Eötvös Lóránd University, Faculty of Education and Psychology, Budapest, 1064, Hungary;

^3^ Messerli Research Institute, University of Veterinary Medicine Vienna, Medical University of Vienna, University of Vienna, Vienna 1210, Austria;

^4^ Department of Philosophy, York University, Toronto, ON M5R 2M8, Canada;

^5^ School of Psychology and Neuroscience, University of St Andrews, St Andrews, KY16 9JP, United Kingdom;

^6^ Max Planck Institute for Evolutionary Anthropology, Leipzig, 04103, Germany.

***** Corresponding author. Email: [hanna.marno@gmail.com](mailto:hanna.marno@gmail.com). ORCID: 0000-0003-2509-267X

**This PDF file includes:**

Figure S1

Figure S2

Tables S1 to S2

Legend for Dataset S1

**Other supplementary materials for this manuscript include the following:**

Dataset S1

Apparatus

The food dispenser device we used was the same device that was used as the ‘blicket detector’ in the study of Völter et al. (2016) (Fig. S1). It consisted of a grey box (30 x 42 cm) with an L-shaped, transparent Plexiglas tube (diameter 7 cm, 12 - 14 cm) attached on top that was turned toward the subjects. Via this tube, subjects could insert the chosen objects into the apparatus. On the other side of the device, facing the experimenter, there was a hole providing access for the experimenter into a chamber inside the apparatus. Inserted objects from the tube fell down into this chamber (Fig. S2). The subjects could see what was inside the chamber as the front side of the chamber, facing the subjects, was made out of transparent Plexiglas (16 x 16 cm). Underneath the chamber, there was a hole (diameter 6 cm) in the box where the food was delivered upon activation of the detector. There were three, white LEDs (diameter: 2.3 cm, 12 V), one integrated in the left sidewall of the chamber, the other two were integrated in the front side of the apparatus. Out of the subjects’ view, there was a hatch inside the apparatus on top of which the reward was placed. The hatch was controlled by an electromagnet (diameter 20 mm, 15 mm, 60 N, 12 V). Moreover, there was a buzzer (75 dB, 400 Hz, 12 V) inside the apparatus that served as acoustic signal. The experimenters could control the detector via a foot pedal that was hidden behind a screen. When they pressed the pedal the detector switched on, i.e. the three LEDs illuminated, the buzzer rang out, and the hatch with the reward was released. The released food (a dry food pellet) rolled down a ramp and stopped in front of the food hole where the subject could see and access it. We used 8 objects of different shapes and colors (Fig. S1). The average size of the objects was 4 - 4 cm. The objects were divided into pairs for the different trials.

Procedure

Subjects were tested individually in the testing rooms of the Wolfgang Köhler Primate Research Center (WKPRC) in Leipzig, Germany. The blicket detector was fixed to the mesh of the test enclosure. Perpendicularly to the blicket detector there was a table, where the experimenters placed the two objects at the beginning of each trial. A Plexiglas panel with two small, circular holes (6 cm) on opposite sides prevented subjects from grabbing the objects.

Only-Ostensive Condition

In the Only-Ostensive condition both demonstrators performed the same actions, except that for the successful demonstrator after the insertion of the object the device gave a short tone, accompanied by lights and the release of a small food pellet, whereas for the unsuccessful demonstrator upon the insertion of the object nothing happened. The demonstrations started by first picking up one of the two objects from the table, making an eye-contact with the subject, saying ‘Hello’ and holding the object in the visibility of the subject. Once the subject was paying attention to the demonstration, the demonstrator approached the device, baited it and put the object on the top of it, said again ‘Hello’ and clapped with her hands, and then for 2 seconds held again the object in the visibility of the subject. After that, she inserted the object in thee device, waited for 3 seconds, and then she took out the object from the device and put it back on the top. Then she repeated again the demonstration starting with saying ‘Hello’, clapping with her hands, showing the object in the visibility of the subject and then inserting again into the device. Upon retrieving the object from the device after the second demonstration, she went back to the table and placed the object at the original position, and then stood in the corner. Then the second demonstrator approached the table and performed the same action sequence. Once both demonstrators finished their demonstrations, they both approached the table on the two sides and pushed the two objects simultaneously towards the two holes of the Plexiglas while they avoided making any eye-contact with the subject. On both trials, the demonstrators used different objects and on both trials the same demonstrator was standing either on the right or on the left side of the table. However, in order to counterbalance the order of successful vs. unsuccessful trials, while on the first trial demonstrator A was successful in activating the device, on the second trial demonstrator B managed to activate the device.

Only-Non-Ostensive Condition

Similar to the Only-Ostensive condition, also in the Only-Non-Ostensive condition both demonstrators performed the same actions, except that one was successfully activating the device, whereas the other did not manage to activate it. The demonstrations started by first picking up one of the two objects from the table, holding it in the visibility of the ape, and saying ‘Aha’ while looking at the object. Once the demonstrator was certain that the subject was paying attention to the demonstration, she approached the device, baited it and put the object on the top of it, said again ‘Aha’ knocked the floor twice with the object, and then for 2 seconds held again it in the visibility of the subject without making an eye-contact with her. After that, she inserted the object in thee device, waited for 3 seconds, and then she took out the object from the device and put it back on the top. Then she repeated again the demonstration starting with saying ‘Aha’, knocking the floor, showing the object in the visibility of the subject and then inserting again into the device. Upon retrieving the object from the device after the second demonstration, she went back to the table and placed the object at the original position, and then stood in the corner. Then the second demonstrator approached the table and performed the same action sequence. Once both demonstrators finished their demonstrations, they both approached the table on the two sides and pushed the two objects simultaneously towards the two holes of the plexiglass while they avoided making any eye-contact with the subject. Just like in the Only-Ostensive condition, on both trials the demonstrators used different objects and on both trials the same demonstrator was standing either on the right or on the left side of the table, but being successful only on one of the trials.

Since during this condition the demonstrator could not see whether the ape was paying attention (because they never looked at the ape), the other demonstrator, who was just passively standing in the room, watched the subject and signaled to the active demonstrator with a gentle cough to start to operate on the device once the ape was attentive.

Ostensive vs. Effective Condition

In the Ostensive vs. Effective condition one demonstrator performed her action in the same manner as the demonstrations were performed in the Only-Ostensive condition, whereas the other demonstrator performed her actions in the same manner as the demonstrations were performed in the Only-Non-Ostensive condition. However, while the non-ostensive demonstrator was successful in activating the device on both trials, the ostensive demonstrator always failed to active it. Furthermore, since we wanted to avoid any potential ‘carry-over effect’ of ostension, i.e. that subjects would generalize the use of ostensive cues and perceive a demonstrator as being ostensive all the time because in a previous trial she used ostensive cues, in this condition the same demonstrator was effective on both trials. In this way, in order to keep a counterbalanced order of successful demonstrations, the first trial started by demonstrator A starting her demonstration, whereas the second trial started by demonstrator B starting her demonstration.

Analysis

Since we wanted to be sure that they understood the causal effect between inserting the object and the outcome, we decided to analyze only those trials when after choosing an object the ape also inserted the object into the device. Non-insertion only happened in one trial during the Only-Non-Ostensive condition, therefore we excluded the data of that trial. We scored whether subjects chose the effective object (codes as “1”) and the ineffective object (coded as “0”). To test whether the apes performed significantly better than the hypothetical chance level of 0.5 we conducted one-sample t-tests (one-tailed). We used a Generalized Linear Mixed Model (GLMM; (Mácha & Baayen, 2008) with binomial error structure and logit link function (McCullagh & Nelder, 1989) to analyze apes’ choices of the effective objects (Table S2). In the model, we included all subjects who completed all trials in all three conditions (N = 18). We included the predictor variable condition (reference category: Communicative vs. Effective condition) and trial number within condition and species as control variables. We also included the interaction between species and condition to examine potential species differences across conditions but removed it from the model given that the interaction did not turn out as significant effect. We included subject ID as a random effect and the random slope components of condition and trial number (Bates et al., 2014). In secondary analyses, we also included rearing history as a factor to the model and added the random intercept of the identity of the effective experimenter.

We determined variance inflation factors (Field, 2013) for standard linear models excluding the random effects using the R package car (Fox, 2011). Collinearity was no issue (all Variance Inflation Factors (VIF) = 1). We assessed model stability by comparing the estimates derived from the model based on all data with those obtained from models with individual subjects excluded one at a time. This revealed the model to be stable with regard to the predictor variable.

P values for the individual effects were based on likelihood ratio tests comparing the full with respective reduced models ((Barr et al., 2013); R function drop1 with argument 'test' set to "Chisq"). The model was implemented in R (version 3.3.2; (Team, 2014)) using the function glmer of the R package lme4 [36]. Confidence intervals for the binomial models were derived using the function bootMer of the R package lme4, using 1,000 parametric bootstraps and bootstrapping over the random effects.

Assessment of looking time data

We recorded the testing sessions by using two cameras from two different perspectives, one was facing the back of the food dispenser device (that is, facing the subject when standing in front of the food dispenser during the demonstrations), and the other was facing the tray where the objects were placed at the beginning of the demonstration and later, during the test, when subjects had to choose one object. We used these two recordings of the two perspectives in order to be able to assess apes’ looking time continuously throughout the entire testing sessions.

The first window of the cue presentation started when the demonstrator first established an eye-contact with the ape and said ‘Hello’ (in the Ostensive condition) or looked at the object and said ‘Aha’ (in the Non-Ostensive condition) and ended when the demonstrator inserted the object into the device, which was the point when the first time window of the Demonstration phase started. The Demonstration phase ended when after the first insertion of the object the device gave a buzzing sound and a food pellet was either released or not, depending on the trial. Then the second time window of the Cue phase started when the demonstrator either clapped with his/her hands and held the object in the visibility of the ape (in the Ostensive condition) or knocked the floor with the object and then held it in the visibility of the ape (in the Effective condition) and ended again with the insertion of the object into the device, at which point the second window of the Demonstration phase started. The second window of the Demonstration phase ended again when after the second insertion of the object the device gave a buzzing sound and a food pellet was either released or not, depending on the type of the trial.

Apes’ looking time was defined by the amount of time they spent with visually fixating in a certain direction or at a certain object. The direction of these fixations was defined by the posture of eyes and the head of the ape in the recordings. The coding was done by two independent coders, one of them was blind to the hypothesis of the study. Inter-coder reliability was 0.98 (Cohen's Kappa).

References

Barr, D. J., Levy, R., Scheepers, C., & Tily, H. J. (2013). Random effects structure for confirmatory hypothesis testing: Keep it maximal. *Journal of memory and language*, *68*(3), 255-278. ttp://dx.doi.org/10.1016/j.jml.2012.11.001

Bates, D., Mächler, M., Bolker, B., & Walker, S. (2014). Fitting linear mixed-effects models using lme4. *arXiv preprint arXiv:1406.5823*.

Field, A. (2013). *Discovering statistics using IBM SPSS statistics*. sage.

Fox, J., & Weisberg, S. (2011). Multivariate linear models in R. *An R Companion to Applied Regression. Los Angeles: Thousand Oaks*.

Mácha, J. (2012). Baayen, RH: Analyzing Linguistic Data A Practical Introduction to Statistics using R. Cambridge University Press, New York, 2008. *Korpus-gramatika-axiologie*, (6), 58-61.

McCullagh, P., & Nelder, J. A. (1989). Generalized Linear Models 2nd Edition Chapman and Hall. *London, UK*.

Team, R. C. (2014). R Core Team. R: A language and environment for statistical computing. R Foundation for Statistical Computing 2014, Vienna, Austria.

Völter, C. J., Sentís, I., & Call, J. (2016). Great apes and children infer causal relations from patterns of variation and covariation. *Cognition*, *155*, 30-43.

<http://dx.doi.org/10.1016/j.cognition.2016.06.009>


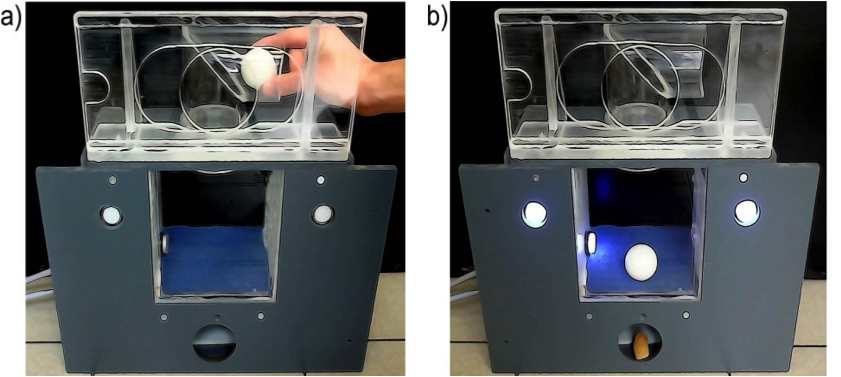


Figure S1. The food dispenser device, before inserting the object (a) and upon successful activation (b).

Figure S2. The objects used during the demonstrations to activate the device.

Table S1. Participant list.

| Subject | Species | Sex | Age | Rearing history | Only-Ostensive Condition | Only-Non-Ostensive Condition | Ostensive vs. Effective Condition |
| --- | --- | --- | --- | --- | --- | --- | --- |
| Bangolo | Chimpanzee | Male | 8 | Parent | Yes | Yes | Yes |
| Dorien | Chimpanzee | Female | 37 | Hand reared | Yes | Yes | Yes |
| Fraukje | Chimpanzee | Female | 41 | Hand reared | Yes | Yes | Yes |
| Frodo | Chimpanzee | Male | 23 | Parent | Yes | Yes | Yes |
| Kofi | Chimpanzee | Male | 12 | Parent | Yes | Yes | Yes |
| Lobo | Chimpanzee | Male | 13 | Parent | Yes | Yes | Yes |
| Riet | Chimpanzee | Female | 40 | Hand reared | Yes | Yes | Yes |
| Sandra | Chimpanzee | Female | 24 | Parent | Yes | Yes | Yes |
| Lome | Chimpanzee | Male | 16 | Parent | Yes | Yes | Yes |
| Swela | Chimpanzee | Female | 22 | Parent | Yes | Yes | Yes |
| Tai | Chimpanzee | Female | 15 | Parent | Yes | Yes | Yes |
| Natascha | Chimpanzee | Female | 37 | Hand reared | Yes | Yes | Yes |
| Dokana | Orangutan | Female | 28 | Parent | Yes | Yes | Yes |
| Padana | Orangutan | Female | 20 | Parent | Yes | Yes | Yes |
| Pini | Orangutan | Female | 29 | Parent | Yes | Yes | Yes |
| Raja | Orangutan | Female | 14 | Parent | Yes | Yes | Yes |
| Suaq | Orangutan | Male | 8 | Parent | Yes | No | No |
| Tanah | Orangutan | Female | 8 | Parent | Yes | Yes | Yes |
| Gemena | Bonobo | Female | 12 | Parent | Yes | Yes | Yes |
| Jasongo | Bonobo | Male | 27 | Parent | Yes | Yes | Yes |
| Kuno | Bonobo | Male | 20 | Hand reared | Yes | Yes | Yes |
| Lexi | Bonobo | Female | 18 | Hand reared | Yes | Yes | Yes |
| Luiza | Bonobo | Female | 12 | Parent | Yes | Yes | Yes |
| Yasa | Bonobo | Female | 20 | Parent | Yes | Yes | Yes |

Table S2. Results of GLMM 01: Correct choices across the different conditions

|  | Estimate | SE | χ2 | df | p | 95% CI | |
| --- | --- | --- | --- | --- | --- | --- | --- |
| (Intercept) | 0.387 | 0.912 |  |  |  | -2.254 | 3.546 |
| Condition^1^ |  |  | 7.190 | 2 | 0.027 |  |  |
| Condition^1^: Only-Non-Ostensive | 1.148 | 0.658 |  |  |  | -0.085 | 5.899 |
| Condition^1^: Only-Ostensive | 2.013 | 1.135 |  |  |  | 0.374 | 12.189 |
| Trial number | -0.455 | 0.598 | 0.625 | 1 | 0.429 | -2.883 | 0.826 |
| Species^2^ |  |  | 1.023 | 2 | 0.600 |  |  |
| Species^2^: chimpanzees | -0.437 | 0.617 |  |  |  | -2.696 | 1.081 |
| Species^2^: orangutans | 0.268 | 0.904 |  |  |  | -1.835 | 3.093 |

Notes: Reference categories: ^1^ Ostensive vs. Effective condition, ²bonobos.

Table S3. Results of GLMM 02 (secondary analysis with rearing history included as a factor): Correct choices across the different conditions

|  | Estimate | SE | χ2 | df | p | 95% CI | |
| --- | --- | --- | --- | --- | --- | --- | --- |
| (Intercept) | 0.56 | 1.02 |  |  |  | -3.53 | 9.78 |
| Condition^1^ |  |  | 7.34 | 2 | 0.026 |  |  |
| Condition^1^: Only-Non- Ostensive | 1.16 | 0.67 |  |  |  | -0.04 | 17.20 |
| Condition^1^: Only-Ostensive | 2.11 | 1.24 |  |  |  | 0.47 | 24.03 |
| Rearing history^2^ | -0.30 | 0.73 | 0.15 | 1 | 0.703 | -3.95 | 2.38 |
| Trial number | -0.45 | 0.60 | 0.59 | 1 | 0.442 | -9.12 | 1.24 |
| Species^3^ |  |  | 1.13 | 2 | 0.567 |  |  |
| Species^3^: chimpanzees | -0.43 | 0.62 |  |  |  | -3.62 | 1.65 |
| Species^3^: orangutans | 0.37 | 0.95 |  |  |  | -2.45 | 7.79 |

Notes: Reference categories: ^1^ Ostensive vs. Effective condition, ²hand-reared, ^3^bonobos.

Table S4. Results of GLMM 03 (secondary analysis with ID of effective experimenter included as a random factor): Correct choices across the different conditions

|  | Estimate | SE | χ2 | df | p | 95% CI | |
| --- | --- | --- | --- | --- | --- | --- | --- |
| (Intercept) | 0.37 | 0.91 |  |  |  | -4.92 | 9.31 |
| Condition^1^ |  |  | 5.61 | 2 | 0.061 |  |  |
| Condition^1^: Only Non- Ostensive | 1.14 | 0.65 |  |  |  | -0.03 | 18.36 |
| Condition^1^: Only Ostensive | 1.99 | 1.13 |  |  |  | 0.48 | 25.55 |
| Trial number | -0.44 | 0.59 | 0.60 | 1 | 0.440 | -8.52 | 1.10 |
| Species^2^ |  |  | 0.99 | 2 | 0.609 |  |  |
| Species^2^: chimpanzees | -0.44 | 0.61 |  |  |  | -5.02 | 1.28 |
| Species^2^: orangutans | 0.23 | 0.87 |  |  |  | -2.94 | 3.97 |

Notes: Reference categories: ^1^Ostensive vs. Effective condition, ² bonobos.

Dataset S1. Apes’ object choices and looking time data in the three (Only Ostensive, Only Non-Ostensive and Ostensive vs. Effective) conditions.
